# Impact of Digitalization in Dentistry on Technostress, Mental Health, and Job Satisfaction: A Quantitative Study

**DOI:** 10.3390/healthcare13010072

**Published:** 2025-01-03

**Authors:** Monika Bernburg, Julia Sofie Gebhardt, David A. Groneberg, Stefanie Mache

**Affiliations:** 1 Institute for Occupational and Maritime Medicine (ZfAM), University Medical Center Hamburg-Eppendorf (UKE), Seewartenstraße 10, 20459 Hamburg, Germany; 2 Institute of Occupational, Social and Environmental Medicine, Goethe University, Theodor-Stern-Kai 7, 60509 Frankfurt am Main, Germany

**Keywords:** digitization, dentistry medicine, dentists, digital stress, medical staff, practices, preventive measures, technostress

## Abstract

**Background:** The utilization of digital technologies in the field of dentistry is becoming increasingly prevalent. Such technologies facilitate more precise and efficient dental treatment while also enhancing the overall quality of care. The advent of digitalization has brought with it a plethora of advantages, yet it has also given rise to a number of potential challenges. These have the potential to give rise to a variety of negative consequences, including an increase in stress perception. **Objectives:** This study identifies the digital demands and resources as well as the prevalence of digital stress perception among German dentists. Furthermore, the study examines the relationship between digital stress perception and work- and health-related outcomes, and it identifies potential preventive measures. **Methods:** The quantitative cross-sectional study involved a total of 325 German dentists. Data collection took place between January and April 2024. The questionnaire was validated using several established scales, including the Technostress Scale and the Copenhagen Psychosocial Questionnaire (COPSOQ). Multiple correlation and regression analyses were conducted to ascertain the reliability and validity of the data collected. **Results:** The study results demonstrated that the participating dentists exhibited a moderate level of digital stress (M = 3.73 (SD = 0.71). Regarding the individual technostress creators, the highest mean was observed for the constructs of techno-overload (M = 3.91; SD = 0.76), techno-complexity (M = 3.63; SD = 0.71), and techno-uncertainty (M = 2.01; SD = 0.75). The participants reported an average level of exhaustion symptoms (M = 3.21; SD = 0.91) and job satisfaction (M = 4.52; SD = 0.78). The association between technostress and emotional exhaustion (as a burnout symptom) showed a significant positive correlation (r = 0.38; CI: 0.07, 0.52; *p* < 0.05). A significant negative correlation was observed between the variables of technostress and job satisfaction, with a correlation coefficient of r = −0.33 (CI: −0.25, 0.07; *p* < 0.05). **Conclusions:** This study presents preliminary findings on the digital stress experience in dentistry and relevant associations. In the context of ongoing digitalization, there is a need for support and preventive measures to reduce technology-related stress. An optimized design of digital applications and the working environment are of crucial importance to improve the health of dentists and the quality of patient care.

## 1. Introduction

The utilization of digital technologies and tools to enhance the provision of dental services is defined as the digitalization of dental care [1]. Digitalization in dentistry refers to the use of advanced technologies such as computer-aided design and computer-aided manufacturing (CAD/CAM) to enhance various aspects of dental care [2]. The introduction of digital technologies, such as digital X-ray techniques, CAD/CAM systems and electronic patient records, has significantly changed the way dental practices work. Studies show that these technologies bring both benefits and challenges.

These technologies enable dentists to capture highly detailed digital impressions of the teeth and surrounding tissues, which can then be used to create precise and customized restorations such as crowns, bridges, and dentures [1,2]. Digitalization in dentistry has revolutionized the field, making procedures more efficient, accurate, and comfortable for both patients and dentists [3,4,5]. Furthermore, digitalization in dentistry has also improved communication between dental laboratories and dental offices, as digital files can be easily shared and accessed [6]. This has resulted in faster turnaround times for restorations and greater collaboration among dental professionals. Digital imaging and analysis tools help dentists to make more accurate diagnoses and design treatment plans more effectively [7]. The use of digitalization in dentistry has transformed the way dental care is delivered, providing greater precision, efficiency, and patient comfort. By eliminating the need for traditional impression materials and manual labor, digitalization in dentistry saves time and reduces errors [8].

In addition, efficiency can be increased by using automated processes, such as appointment scheduling and patient management, which reduce the administrative burden and allow the practice team to focus on patient care [3,4,5]. Digital assistance programs allow quick access to patient records and treatment histories, which improves decision-making [6].

Overall, digitalization in dentistry has had a significant impact on improving the quality of care provided to patients and streamlining the workflow in dental practices [2,9]. By using digital technologies, dentists can provide more precise and personalized treatments, leading to better treatment outcomes and quality [10].

While the implementation of new technologies may enhance efficiency and precision, it is also possible that adapting to these changes may result in stress and uncertainty for dental professionals. The implementation of digital systems can involve high investments, both in terms of software and the necessary hardware. Dentists and practice teams can become dependent on technology, which can lead to problems if systems break down or do not work properly [6]. The storage and processing of patient data in digital systems harbors risks in terms of data protection and cyber-attacks [11]. The digital (working) environment may contribute to cognitive load and, consequently, an elevated risk of information overload [12,13,14]. Therefore, it is becoming more crucial to consider the impact of digitization processes to gain a deeper understanding of the ways in which relevant stressors and resources interact in this setting.

Research findings identify specific stressors associated with the use of digital technologies in medical care. These include technical problems, inadequate training in the use of new systems and the pressure to keep up with rapid technological developments [11,12]. The introduction of new digital systems often requires extensive training for staff, which can take up time and resources [6]. A study by Gallager et al. (2021) emphasizes that dentists often suffer from stress when they must deal with new digital tools [12].

A few studies have shown that dentists are at higher risk of mental health problems, including stress perception and burnout [15,16]. Digital transformation can exacerbate these problems, especially if dentists feel that they are not adequately prepared for the new demands. Some studies have looked at the coping mechanisms that medicals develop to deal with digital stress. These include seeking support from colleagues, participating in further training and implementing stress management strategies. Studies show that dentists often use informal networks to share experiences and find solutions to digital challenges [17,18].

To summarize, the extant scientific evidence on technostress, in general, has already demonstrated elevated stress levels among employees engaged in work involving digital technologies. Furthermore, this evidence has identified a range of factors that contribute to technostress, as well as those that serve to mitigate its effects [19]. Moreover, these studies have provided insights into the prevalence of digital stress perception and the associated consequences of digitalization [20]. However, research on digital stress in dentistry, particularly among dentists, is still in its infancy. Given the dearth of research in this field, further investigation is highly warranted.

### 1.1. Theoretical Framework

In order to gain a clarifying insight into the relationship between digital stressors and the associated effects on mental health, both the Technostress model and the Job Demands-Resources (JD-R) model were employed in this study [21,22].

In recent decades, the phenomenon of digital stress, also known as “technostress”, has gained prominence and is currently a topic of growing interest in academic research. The concept of technostress was initially proposed by Brod in 1984, who demonstrated that the utilization of digital technologies in routine occupational contexts does not solely encompass beneficial outcomes but may also give rise to a spectrum of adverse emotional states, culminating in the emergence of stress. This theoretical framework, also known as the Conceptual Model for Understanding Technostress, was subsequently developed by Ragu-Nathan et al. (2008). Ragu-Nathan et al. define technostress as “stress experienced by end users in organizations as a result of their use of ICTs” [17]. The model was developed in response to the need for an instrument to assess exposure to digital stressors and the level of persisting technostress, with the aim of improving understanding of the stresses and resource situation of employees at work in general. Accordingly, Ragu-Nathan and his research team developed and empirically validated a questionnaire comprising items pertaining to two principal constructs: the “technostress creators”, or digitisation-related stress factors, and the “technostress inhibitors”, which correspond to digitisation-related resources or protective factors [17].

Ragu-Nathan et al. distinguish between the causes of technostress, referred to as digital stressors, and technostress inhibitors, which are also called digital resources or protective factors, which exert a significant influence on the occurrence of stress [23]. The researchers identified five key factors within the Technostress Creators construct, which are outlined below: The identified factors were as follows: “techno-overload”, “techno-invasion”, “techno-complexity”, “techno-insecurity” and “techno-uncertainty”. Additionally, the authors identified the “technostress inhibitor” factors of “literacy facilitation”, “technical support provision”, “involvement facilitation”, “job satisfaction”, “organizational commitment”, and “continuance commitment” [23].

### 1.2. Objectives

The study was designed to address the knowledge gap regarding the perception of technostress in the context of dentistry. It aimed to identify the factors that contribute to technostress, as well as those that mitigate it, and to examine the relationship between technostress and mental health, as well as job satisfaction. The present study is guided by several objectives. The study aims to: 1. document the utilization of digital applications in the context of dental care, and 2. examine the relationship between the use of digital applications and the perception of technostress, as well as the effects on health and work-related outcomes. Furthermore, this study aims to identify the stressors and resources resulting from the utilization of digital technologies in the field of dentistry. The following hypotheses are put forth for consideration:

**Hypothesis** **1.** *A significant negative correlation exists between dentists’ levels of perceived technostress and their subjective ratings of the usefulness and ease of use of the digital technologies they utilize*.

**Hypothesis** **2.** *There is (a.) a significant positive association between levels of technostress creators and levels of perceived technostress among dentists and their mental health symptoms. Conversely, there is (b.) a significant negative relationship between these factors and job satisfaction*.

**Hypothesis** **3.** *There are significant associations between levels of technostress-inhibitors among dentists and (a.) levels of mental-health symptoms, (b.) levels of job satisfaction*.

**Hypothesis** **4.** *The prevalence of technostress is markedly diminished among dentists whose employers proactively implement comprehensive preventive measures, such as the provision of information and training*.

## 2. Materials and Methods

### 2.1. Study Design and Data Collection

A quantitative cross-sectional study was conducted via an online survey in German dental practices. To participate in the study, dentists must currently be employed in a dental practice or run a dental practice independently. A further criterion was that the study participants must use digital technologies in their practice, such as the electronic patient file or a digital application. This means that the dental practice must have introduced at least one digital technology.

The requisite sample size was determined through the utilization of G*Power software (version 3.1.9.7), with an effect size of ƒ2 = 0.15 (classified as a medium effect), an alpha level of 0.05, the inclusion of predictors, and a statistical power of β = 0.80. This resulted in a total of at least 103 participants being necessary for the study.

The online survey was conducted over a period of two months, commencing in January 2024. The pertinent dental practices were identified based on the results of an analysis of several internet portals. Subsequently, the study participants were initially contacted via email or telephone. Following a three-week period, reminder letters were dispatched to all dentists.

### 2.2. Measures

The construct of technostress was operationalized as a set of job demands, while the construct of technostress inhibitors was operationalized as a set of job resources. Two outcome variables were assessed: burnout and job satisfaction.

#### 2.2.1. Sociodemographic Variables

The initial section of the questionnaire was designed to gather information pertaining to the socio-demographic characteristics of the respondents. This included data on their professional status, utilization of digital applications, age, gender, the regional affiliation of their dental practice, their experience as a dentist, and their typical weekly working hours.

#### 2.2.2. Frequency of Use and Attitudes Towards Digital Technologies in Dental Care

The frequency and duration of utilization of digital technologies, in addition to the attitudes held towards said technologies, were documented. Two items, developed by the researchers themselves, were used to record the frequency and duration of use. Furthermore, the two validated construct scales, “Perceived Usefulness (PU)” and “Perceived Ease of Use (PEOU)”, from the German version of the Technology Acceptance Model (TAM), were employed to assess attitudes towards the technologies under investigation [24]. The Cronbach’s alpha values for the perceived usefulness (PU) scale and the perceived ease of use (PEOU) scale were 0.85 and 0.79, respectively, indicating that the instruments exhibited satisfactory reliability.

#### 2.2.3. Techno-Stressors and Technology-Associated Resources

Subsequently, the incidence of digital stressors in the workplace is documented. An adapted version of the standardized and validated ‘Technostress’ scale, as proposed by Ragu-Nathan et al. (2008), was selected for this purpose. The “technostress” dimensions were surveyed: “techno-overload”, “techno-complexity” and “techno-uncertainty” in the German version [19]. Overall, this instrument has satisfactory reliability values for the various dimensions and good discriminant and convergent validity without significant error correlations between the included items [23].

In order to gain a more nuanced understanding of the stressors, a self-developed item based on an item from the HIMSS study (2015) was also introduced [25].

The two technostress inhibitor constructs, namely “literacy facilitation” and “involvement facilitation”, from the Technostress Scale by Ragu-Nathan et al. (2008), were also employed (9 items) [23].

Furthermore, dentists were invited to provide additional insights on techno-stressors and technostress-inhibiting factors in the dental practice through free-text responses, with the aim of gaining a more comprehensive understanding of the stressors and resources at hand.

#### 2.2.4. Preventive Measures in Dealing with Technostress

A further set of questions pertains to the implementation of health-promoting strategies for the management of digital stress (Likert-scale with 8 items in total) [26,27]. In addition, a self-developed scale was used to assess the subjectively perceived effectiveness of existing preventive measures in dealing with technostress. The participating dentists were invited to provide examples of preventive measures that they had implemented in order to reduce digital stress in dentistry. These examples were requested in free-text responses. The measures mentioned in the free-text responses were then categorized and quantified as follows.

The variables pertaining to preventive measures were classified into three categories. The respondents were invited to indicate their level of disagreement, partial agreement or agreement with the aforementioned statements by selecting one of the following response options: “I disagree” (corresponding to a low level of measures implemented), “I partly agree” (corresponding to a medium level of measures implemented) and “I agree” (corresponding to a high level of measures implemented).

#### 2.2.5. Analysis of Burnout and Job Satisfaction as Health and Work-Related Outcomes

The assessment of emotional exhaustion as a symptom of the “burnout construct” was conducted using the standardized and validated short scale of the COPSOQ (2022), which has been previously validated [25]. This comprises three items. Moreover, the construct of job satisfaction was evaluated using the abbreviated scale developed by Ragu-Nathan et al. (2008), comprising three items [23]. All scales demonstrated satisfactory quality criteria in terms of reliability and validity. The Cronbach alpha coefficient ranged from 0.81 to 0.85. Furthermore, free-text responses to the question of which health effects are perceived due to technostress were subjected to analysis.

#### 2.2.6. Research Data Analysis

The dataset was initially subjected to a thorough examination to identify any potential missing values and to ascertain its overall plausibility. The Shapiro–Wilk test, along with measures of skewness and kurtosis, and the use of histograms, were employed for the purpose of evaluating the distribution of the data. In the event that the requisite normal distribution of the continuous variables was not satisfied, the correlations were analyzed using the bootstrapping method. Additionally, multiple regression analyses were conducted. Furthermore, parametric tests, specifically *t*-test and ANOVA, were employed when the data exhibited a normal distribution. The alpha level was set at 0.05 or less. Furthermore, correlation analyses were conducted using the Pearson correlation coefficient for continuous variables and the Spearman’s rho correlation coefficient for ordinal variables. IBM SPSS version 29 statistical software was used. Qualitative content analyses were conducted to examine the free-text responses.

## 3. Results

### 3.1. Sample Description

A total of 368 dentists participated in the online survey. Following the application of missing value and plausibility checks, 43 questionnaires were excluded. The majority of participating physicians were male (58.7%; n = 191) (see Table 1). Additionally, 75.1% of the respondents were self-employed. The majority of respondents had been working in dentistry for over a decade, with 78% having been in practice for more than ten years.

### 3.2. Descriptive Statistical Analysis

#### 3.2.1. Digital Technologies’ Frequency and Duration of Use

When queried about their frequency of use, the majority of participating dentists indicated that they would utilize digital technologies for several hours each day (98.9%; n = 325) (see Table 2).

#### 3.2.2. Techno-Stressors in Dentistry Practice

The overall level of technostress was found to be at a medium level for all participants (M = 3.73; SD = 0.71 (1 = strongly disagree/no technostress; 5 = strongly agree/high technostress)). Regarding the individual technostress creators, the highest mean was observed for the constructs of techno-overload (M = 3.91; SD = 0.76), techno-complexity (M = 3.63; SD = 0.71), and techno-uncertainty (M = 2.01; SD = 0.75).

Furthermore, several potentially deleterious side effects or stressful elements were investigated.

As a result of the influence of techno-stressors, the greatest level of stress was reported by 29.6% of dentists (n = 325) in relation to the issue of documentation. Subsequently, technical system errors were identified as a source of stress for 27.7% of respondents. A total of 57% of participants indicated that they experience stress because of the dual documentation aspect of the technology, with a frequency rating of “often” or “very often”. A significant proportion of respondents (27%) identified a lack of data security as a potential issue. Technostress-promoting factors in dentistry can include various aspects that increase the stress caused through the use of digital technologies. Table 3 presents a selection of the most frequently cited factors by dentists in the free-response section.

#### 3.2.3. Technostress Inhibitors and Resources

The mean of the overall expression of the technostress inhibitors was calculated as M = 2.91 (SD = 0.75). The expression of the technostress inhibitors for facilitating reading and writing was M = 3.31 (SD = 1.07), whereas the expression of the inhibitors for facilitating participation was M = 1.89 (SD = 0.82).

Technostress-inhibiting factors in dentistry can help to reduce the stress caused by the use of digital assistance systems. Factors mentioned by the dentists in free answer formats were categorized (see Table 4).

#### 3.2.4. Health-Promoting Measures for Dealing with Technostress

The implementation of health promotion measures was observed to a limited extent, with a mean score of 2.51 (SD = 0.79). The most prevalent measures were training and skills acquisition, with a mean value of 3.19 (SD = 1.05). The necessity for supplementary qualifications was assessed as being of significant importance. The participating dentists provided examples of preventive measures that have been implemented to reduce digital stress in dentistry. The measures provided in the free-text responses were categorized and quantified as follows:

1. Technological support/utilization of IT support services (n = 218);
2. The implementation of regular training programs for dental personnel on the utilization of digital technologies (n = 185);
3. The selection of intuitive and user-friendly software solutions (n = 108);
4. The utilization of digital communication tools (such as patient portals) (n = 49);
5. Periodic assessment of digital tools/Implementation of periodic reviews and adaptations of the technologies employed (n = 27).

A total of 72% of the dentists indicated that the existing preventive measures had been beneficial. A mere 37% of the participants expressed high levels of satisfaction, while over 45% of the dentists indicated that they were either dissatisfied or highly dissatisfied with the implemented preventive measures.

#### 3.2.5. Technology Acceptance in Dental Practice

Technology acceptance resulted in a mean value of M = 3.89 (SD = 0.82, n = 325) for the construct of perceived usefulness. With regard to the construct scale of perceived ease of use, the survey yielded an overall mean value of M = 3.43 (SD = 0.78, n = 325).

#### 3.2.6. Work- and Mental Health-Related Outcomes

The mean score for burnout symptoms was M = 3.21, with a standard deviation of SD = 0.91. The mean level of job satisfaction was 4.52 (SD = 0.78).

In addition, dentists reported health outcomes. The outcomes provided in the free-text responses were categorized and quantified in the following Table 5.

### 3.3. Analytical Statistical Analysis

The analyses conducted to test our initial hypothesis revealed a moderately negative correlation between the variables of subjectively perceived usefulness (PU) and technostress (r = −0.42; *p* = 0.01) (see Table 6). Furthermore, a moderately negative correlation was observed between the variables of subjectively perceived ease of use (PEOU) and technostress (r = −0.52; *p* = 0.01). Considering the aforementioned findings, it can be concluded that the initial hypothesis is supported.

The results of the multiple regression analysis indicate that the two independent variables, namely subjectively perceived usefulness (PU) and subjectively perceived ease of use (PEOU), respectively, could account for 23% of the variance in the overall expression of technostress (R2 = 0.23, n = 325, *p* < 0.001). The combined influence of the two independent variables was also found to be significant. This suggests that as the perceived usefulness (PU) and perceived ease of use (PEOU) of the relevant digital technologies increased the levels of technostress experienced by employees decreased.

The association between technostress and emotional exhaustion (as a burnout symptom) showed a significant positive correlation (r = 0.38, CI: 0.07, 0.52), as evidenced by the correlation analyses. This finding supports the assumption set forth in Hypothesis 2a. A significant negative correlation was observed between the variables of technostress and job satisfaction, with a correlation coefficient of r = −0.33 (CI: −0.25, 0.07) and a *p*-value less than 0.05. Therefore, Hypothesis H2b can also be accepted.

The multiple regression analysis for the techno-stressor variables and the outcome emotional exhaustion as a burnout symptom demonstrated that the three independent variables, namely techno-overload, techno-complexity, and techno-uncertainty, could each account for 17% of the variance of the dependent variable emotional exhaustion within this model. Furthermore, the result was found to be highly significant (*p* < 0.001). The influence of the predictor technology non-overload was also rated as highly significant (*p* < 0.001). The regression results for the remaining predictor techno-complexity were also statistically significant (*p* < 0.05). The third predictor techno-uncertainty was not significant (*p* > 0.05).

The model demonstrated that 20% of the variance in job satisfaction could be attributed to the three independent variables, namely “techno-overload”, “techno-complexity” and “techno-uncertainty”. The model was found to be statistically significant at the *p* < 0.05 level. In particular, the influence of the predictor “techno-overload” was found to be significantly influential (*p* < 0.05). The results for the influence of techno-complexity were also found to be statistically significant. Nevertheless, no statistically significant correlation was observed between techno-uncertainty and job satisfaction (*p* > 0.05) (see Table 7).

The correlation analyses of the two technostress inhibitor variables, “literacy facilitation” and “involvement facilitation”, and the burnout variable revealed a significant association, with *p* < 0.05.

The correlation analysis between the technostress inhibitors and the variable of job satisfaction and work engagement yielded a small positive result, with a correlation coefficient of r = 0.27 and a *p*-value smaller than 0.05, indicating a statistically significant association; thus, assumptions 3a, b, c can be verified.

In order to test the hypothesis that there are differences in the perception of technostress depending on the prevention measures employed, one-way ANOVAs were conducted, which revealed significant differences in the levels of digital stress experienced by the participants in the different prevention groups. The resulting F-value (df = 2, 325) was 2.284, with a *p*-value less than 0.05. This finding lends support to Hypothesis 4.

## 4. Discussion

The present study examined the perceptions and assessments of German dentists regarding digital stress perception, stressors, and resources in the context of dental care. Additionally, associations were identified between health and work-related aspects. The potential for preventive measures to address digital stress was explored, and their significance for dentists was elucidated.

The dentists surveyed indicated that they utilize digital technologies with considerable frequency. Their use was rated as either acceptable or neutral. Nevertheless, the perceived user-friendliness value was relatively low in comparison to the results of other studies conducted in medical settings [28,29]. This may be indicative of potential issues with the utilization of digital technologies, which warrant further investigation. Nevertheless, these findings are consistent with broader insights on employee perceptions of digital technology and potential barriers to its utilization [6,9,30,31].

The results indicated that dentists reported an average level of technostress, with the highest proportion of techno-overload. This finding is consistent with the general perception of users that technology forces them to work at a faster pace. These findings are consistent with those of other recent studies in the medical field [32,33,34].

### 4.1. Technology-Associated Stressors and Resources

The research findings showed that techno-stressors in dentistry can include various aspects that increase the stress levels of dentists. The constant evolution of dental technologies and equipment can be overwhelming. Dentists need to regularly educate themselves and to invest in new technologies to stay up-o-date with the rapid progress of digitalization and latest developments. Managing patient data, especially in digital systems, can be challenging. As shown in other research formats, data protection regulations and the need to process information quickly and efficiently can create additional pressure [35].

As demonstrated in a study by Golz et al. (2021), several tasks often need to be completed simultaneously, such as treating patients, managing appointments, and communicating with the team [20]. This can lead to stress when demands are high [23]. The use of digital assistance systems entails an increased dependency on technology. When technical problems occur, e.g., equipment or software failures, this can disrupt the workflow and cause additional stress [36]. These findings are also consistent with other research, which suggests that technical issues, a lack of technical expertise, a lack of training and support, or a lack of time are significant barriers to the adoption of technology [37,38].

Our results also show that most dentists lack resources to offset the disadvantages of technology. Moderate levels were measured for resources facilitating literacy, but particularly low for participation. These findings align with research showing a lack of participation resources [31]. Relevant resources included support from colleagues, individual resources, such as digital competence, and support factors, such as effective IT support and operational back-up procedures [32,38]. In a separate study, the following factors were identified as being of significant importance: transparency, the provision of high-quality and sufficient training, the availability of technology providers for the resolution of queries, or the identification of issues, coaching, and peer monitoring [39]. It is also important to highlight the provision of internal technical support as a key factor [40].

### 4.2. Associations Among Perceived Techno-Stressors and Dentists’ Technostress Inhibitors and Mental Health Symptoms and Job Satisfaction

The present results indicate a positive correlation between techno stressors and the perception of mental health symptoms. The results of studies conducted in other medical specialties corroborate the perceived health correlations and yield comparable outcomes, e.g., that the surveyed physicians believe that digital stressors contribute to health symptoms (i.e., burnout) [20,28,41]. These correlations serve to underscore the vital importance and necessity of implementing preventive measures to mitigate the experience of digital stress.

As the present study also found a significant correlation between technostress and the resulting job satisfaction, Hypothesis 2 was also confirmed. This is in line with more recent studies that have shown significant negative correlations between the results of job satisfaction and various technostress factors or the overall level of technostress [42]. The results presented permit the conclusion that the design of framework conditions in the context of digitalisation in dentistry is of great consequence in order to sustainably promote the job satisfaction of dentists.

### 4.3. Associations Between of Preventive Measures and Dentists’ Technostress

Preventing technostress remains crucial to avoid negative effects, especially since resources were scarce. Tests showed significant differences in technostress levels according to measures implemented, with the most significant gaps between those with and without employer measures. The study revealed that dentists with low levels of preventive measure implementation exhibited higher levels of technostress. It is therefore possible to test Hypothesis 4 in the light of these results. A systematic review of the literature on the outcomes of different interventions shows that a combination of different preventive measures can alleviate burnout symptoms caused by digitalization [43]. While the cross-sectional analysis results do not permit a causal conclusion to be drawn, they do indicate the potential relevance of prevention measures in this context. A large number of measures were presented, the effectiveness of which needs to be validated in future studies.

### 4.4. Strengths and Limitations

A strength of the study is the recruitment strategy based on dental practices. Another strength is the use of validated and recognized scales, including the TAM. However, the study has limitations. A significant drawback of this study is its cross-sectional design, which precludes the establishment of causality. Additionally, the use of self-report scales may introduce a social desirability bias, which could potentially influence the results. The sample is not adequately diverse. The sample size was insufficient to permit an analysis of potential differences in technostress levels between specific groups. Improved recruitment strategies are needed to avoid the under-representation of specific groups in surveys, such as junior doctors or female doctors. Future research should consider these aspects in longitudinal or experimental designs.

A small sample size and lack of diversity in the study may limit the representativeness and generalizability of our results. Future studies should, therefore, include more participants.

The questionnaire used relies heavily on self-reported data, which can introduce bias, particularly in sensitive areas such as mental health and burnout. For future research, complementing surveys with objective metrics (e.g., clinical outcomes or system usage logs) would enhance validity.

### 4.5. Implications for Research

Future research on digital stress in dentistry could focus on several key areas to better understand its impact and develop effective solutions. Here are some potential research directions:

It would be beneficial to investigate the prevalence of digital stress among dental professionals and its correlation with long-term mental health issues such as burnout and depression. Studies should also assess the effectiveness of different training models (e.g., workshops, online courses, peer mentoring) on reducing digital stress and improving technology adoption. The present study did not consider regular training and IT support as potential intervention strategies. However, these could be the subject of future studies, in which they could be evaluated in the context of other relevant factors. Furthermore, the impact of continuous learning on the retention of skills and the confidence of individuals in utilizing digital tools could be investigated.

Further research should also investigate the relationship between ergonomic workplace designs and levels of digital stress among dental practitioners. In addition, studies should focus on how team collaboration and communication affect individual experiences of digital stress.

It may also be interesting to investigate how patients perceive digital assistance and how these influence their satisfaction with dental care.

In addition, coping mechanisms should be analyzed in this context. The aim could be to investigate which effective coping strategies dentists can use to deal with digital stress and how these can be promoted in practices. Moreover, the effectiveness of resilience training programs in reducing digital stress and enhancing overall well-being should also be explored.

A further research aspect is artificial intelligence (AI) integration. Hereby, the effects of AI and advanced digital tools on workflow, stress levels, and decision-making processes in dental practices should be studied.

Focusing on these areas will help build a comprehensive understanding of digital stress in dentistry and lead to targeted interventions that enhance the well-being of dental professionals while improving patient care. By addressing the psychological, social, and technological dimensions of digital stress, future research can significantly contribute to a healthier dental workforce.

There is also a lack of knowledge about prevention and experience with it, so more research is needed on effective prevention strategies to counteract technostress. Field studies on different preventive measures are recommended for this.

### 4.6. Implications for Practice

One strategy for mitigating digital stress is to deliberately incorporate digital tools and software into the dental practice that are as intuitive as possible and necessitate minimal training and integration time. It is, thus, possible to develop standard operating procedures (SOPs) to regulate the utilization of digital technologies, with the objective of ensuring consistency and reducing confusion. Furthermore, it is important for successful integration of new technologies that they fit into the practice’s IT structure to ensure a smooth workflow. As our study results show, it is of significant importance to attend training courses on a regular basis in order to remain informed about the latest technological developments and best practices, as well as to cultivate confidence and expertise. An alternative approach to providing support is the appointment of mentors/multipliers within the practice, who can disseminate their knowledge and experience of digital tools. Apart from theoretical expert knowledge, experimenting, observing, and learning by performing help to improve practical skills and to gain confidence in dealing with digital technologies.

It is possible to implement work systems that integrate multiple functions (such as patient records, appointment scheduling, and invoicing) within a single platform. This will result in a reduction in the necessity for data entry and administrative effort. The deployment of secure messaging and telehealth solutions facilitates the expedient resolution of patient queries, thereby reducing the necessity for in-person appointments. Specific work hours can be established, thus, allowing for periods of downtime that may help to reduce burnout and facilitate the establishment of a healthy life–domain balance. It is recommended that scheduled breaks be incorporated into the daily routine to facilitate recharging and mitigate the negative effects of prolonged digital engagement. In addition, delegating responsibilities related to digital tasks, such as data entry or patient communication, can reduce dentists’ workload and provide support for team members. It is further recommended that meetings should be held on a regular basis to discuss challenges and to share strategies for the effective management of digital tools.

The implementation of automated systems for tasks such as appointment reminders, billing, and follow-ups can effectively reduce the manual workload. Moreover, the implementation of AI-based solutions for diagnostic or treatment planning purposes can be regarded as a potential means of streamlining decision-making processes. It is imperative that ongoing assessments of the efficacy of digital tools are conducted and that any necessary optimizations are implemented. In addition, it is feasible to identify prospective avenues for enhancement in digital tools by soliciting feedback from both staff and patients.

## 5. Conclusions

The present study offers valuable insights into the prevalence of digital stress, stressors, and inhibitors in the context of dental care. Furthermore, potential correlations with parameters relevant to work and health were identified. The principal conclusion of this study is that dentists are cognizant of digital stress and its associated factors. The study identified relevant stress factors and the coping strategies that dentists employ in dealing with these technologies. In some cases, these coping strategies have already been utilized.

However, the results of this study also show that even basic preventative measures, such as regular, targeted training, are not adequately implemented in all dental practices. This is particularly noteworthy considering that the dentists in this study had moderate levels of technostress and limited resources. It is well known that doctors are susceptible to negative stress consequences such as burnout due to their exposure to a range of workplace stressors. The implementation of the mentioned support strategies may enable dentists to effectively manage digital stressors and reduce their daily job demands. Prioritizing training, optimizing workflows, and embracing technology can facilitate the creation of a more efficient and less stressful work environment, which ultimately benefits both practitioners and patients.

In light of the current state of research, it is evident that a comprehensive and supportive introduction to, and subsequent monitoring of, the implementation of new digital technologies is of paramount importance. Such measures may include introductory training, ongoing IT support, and the health-promoting management of digital stressors. This will ensure that digitalization processes are as effective and health-promoting as possible and that the full potential of digitalization can be exploited.

Future research should implement intervention studies to determine the effectiveness of preventive measures in this context. This is in addition to a general expansion of research on the current topic to further improve knowledge about the interaction between different work-related stressors, resources, and outcomes.

## Figures and Tables

**Table 1:** Characteristics of study population (n = 325).

Characteristic	Frequency (n)	Percentage (%)
Gender		
Male	191	58.7
Female	133	41.1
Third Gender	1	0.2
Age		
20–29 years	25	7.7
30–39 years	61	18.7
40–49 years	103	31.7
50–59 years	95	29.2
60 years and older	41	12.6
Job position		
Self-employed	244	75.1
Employed	81	24.9
Extent of current employment		
Working full time (≥35 h/week)	210	64.7
Working part time (15–34 h/week)	115	35.3
Overall clinical experience		
<5 years	29	9.1
5–<10 years	38	11.8
10–<15 years	39	12.1
15–<20 years	66	20.3
20–<25 years	73	22.1
≥25 years	80	24.6

**Table 2:** Frequency and duration of usage of digital technologies (n = 325).

Characteristic	Frequency (n)	Percentage (%)
Usage frequency		
Daily usage	321	98.9
Usage several times per week	4	1.1
Usage duration (estimated per day)		
<1 h	6	1.8
1–<2 h	20	6.2
2–<3 h	38	11.6
3–<4 h	69	21.3
4–<5 h	102	31.5
5 h or more	90	27.6

**Table 3:** Categories and quotes from the free-text responses relating to techno-stressors.

Category	Citations
Technical problems (n = 109)	*“If there are lots of technical glitches, software errors or equipment failures, it can really mess up the workflow and cause stress, especially if there isn’t much technical support.”* (P267; Engl. translation of original citation.)
Technological overload (n = 107)	*“…the multitude of digital assistance systems and technologies available can be overwhelming”.* (P302; Engl. translation of original citation.) *“Dentists have to keep up with new devices and software all the time, which can be stressful.”* (P25; Engl. translation of original citation.)
Lack of training (n = 83)	*“Not getting the training or not getting enough training on new tech can make us feel uncertain and frustrated if we don’t know how to use the systems properly”.* (P4; Engl. translation of original citation.)
Data management and data protection (n = 53)	*“Responsibility for the correct management of patient data and compliance with data protection regulations can create additional pressure, especially if the systems are complex.”* (P61; Engl. translation of original citation.)
Patient expectations (n = 58)	*“As patients’ expectations of digital services and technologies rise, dentists may feel the pressure to keep up.”* (P249; Engl. translation of original citation.)
Multitasking requirements (n = 83)	*“It can be really overwhelming and stressful to have to manage multiple tasks at once, such as operating digital devices during treatment and managing patient data.”* (P146; Engl. translation of original citation.)
Lack of integration (n = 25)	*“If digital systems are not well integrated into the existing workflow, this can lead to inefficiencies and frustration.”* (P64; Engl. translation of original citation.)
Competitive pressure (n = 16)	*“The pressure to keep up with other practices that may be using more advanced technologies lead to feelings of inadequacy and stress.”* (P8; Engl. translation of original citation.)

**Table 4:** Categories and quotes from the free-text responses relating to techno-stress inhibitors.

Category	Citations
Regular training and continuing education (n = 108)	*“We need to make sure that dentists and their teams get regular training and keep up with the latest developments in digital technologies. This will help them to feel more confident and reduce any uncertainty.”* (P428; Engl. translation of original citation.)
Technical support (n = 107)	*“Reliable technical support that is quickly available in the event of problems.”* (P278; Engl. translation of original citation.)
User-friendly technologies (n = 81)	*“We’re looking for digital assistance systems that are easy to use and intuitive.”* (P148; Engl. translation of original citation.)
Technological customization (n = 71)	*“Making sure that the tech you use in your dental practice is right for the job can help to reduce stress by making it easier to fit everything into your day.”* (P23; Engl. translation of original citation.)
Teamwork and communication (n = 39)	*“Open communication within the practice team and the promotion of teamwork is important.*”(P261; Engl. translation of original *citation*.) *“Reduce stress by better distributing tasks and solving problems together.”* (P261; Engl. translation of original citation.)
Feedback culture (n = 38)	*“We’re all about giving and receiving feedback to help us spot problems early and find solutions before they become a problem.”* (P11; Engl. translation of original citation.)

**Table 5:** Categories and quotes from the free-text responses relating to health-related outcomes and digital stress.

Category	Citations
Mental stress (n = 219)	*“I find that digital demands can really stress me out, especially when there’s so much information to take in all the time.”* (P302; Engl. translation of original citation.)
Burnout (n = 105)	*“If the technology isn’t working properly, it can cause a lot of stress, which in turn leads to exhaustion.”* (P5; Engl. translation of original citation.) *“I’m feeling pretty tired and my performance has dipped a bit.”* (P118; Engl. translation of original citation.)
Cognitive impairment (n = 84)	*“All this digital stuff is making it hard for me to concentrate. I’m finding it harder to remember things and I’m not as productive as I used to be.”* (P79; Engl. translation of original citation.)
Irritability (n = 71)	*“We all know that digital stressors can make us more irritable and frustrated.”* (P249; Engl. translation of original citation.)
Physical complaints (n = 95)	*“Using devices too much can cause problems with your posture, as well as neck and back pain and eye strain.”* (P279; Engl. translation of original citation.)
Cardiovascular problems (n = 19)	*“It’s been linked that too much digital stress can increase the risk of high blood pressure.”* (P35; Engl. translation of original citation.)

**Table 6:** Pearson correlation coefficients for technostress, PU and PEOU (n = 325).

				Overall Expression of Techno-Stressors	Perceived Usefulness (PU)	Perceived Ease of Use (PEOU)
Overall expression of technostress	Pearson correlation			1	−0.42 **	−0.52 **
	Sig. (2-tailed)				0.000	0.00
	N			325	325	325
	Bootstrap ^1^	Bias		0	−0.005	0.00
		Std. Error		0	0.075	0.08
		95% Confidence Interval	Lower		−0.65	−0.70
			Upper		−0.19	−0.20

** Correlation is significant at the 0.01 level (2-tailed). ^1^ Unless otherwise noted, bootstrap results are based on 1000 bootstrap samples.

**Table 7:** Multiple regression analyses of techno-overload, -complexity, -uncertainty and the outcome variables of burnout, job satisfaction (n = 324).

Predictors	b ^a^	*SE* ^a^	t	*p*
	Outcome of Burnout Symptoms			
Techno-overload	0.56	0.13	4.85	<0.001
Techno-complexity	0.41	0.75	1.594	<0.05
Techno-uncertainty	−0.10	0.04	−1.03	>0.05
	Notation. R^2^ = 0.17 (n = 324, *p* < 0.001).			
	**Outcome of Job Satisfaction**			
Techno-overload	−0.24	0.08	−2.27	<0.05
Techno-complexity	−0.20	0.07	−1.71	<0.05
Techno-uncertainty	0.08	0.05	1.48	>0.05
	Notation. R^2^ = 0.20 (n = 324, *p* < 0.05).			

^a^ Confidence intervals und standard errors per BCa bootstrapping with 1000 BCa samples.

## Data Availability

The datasets analyzed during the current study are not publicly available due to German national data protection regulations but are available from the corresponding author on reasonable request.
